# Autoimmune diseases and autoimmune polyglandular syndromes in adult patients with biopsy-confirmed celiac disease: A prospective study

**DOI:** 10.1097/MD.0000000000051184

**Published:** 2026-10-16

**Authors:** Senem Kuşaksızoğlu Kara, Sema Basat, Çağdaş Kara

**Affiliations:** a Department of Internal Medicine, Bakirköy Dr. Sadi Konuk Training and Research Hospital, Istanbul, Türkiye; b Department of Internal Medicine, Umraniye Training and Research Hospital, Istanbul, Türkiye; c Department of Internal Medicine, Gaziosmanpaşa Training and Research Hospital, Istanbul, Türkiye.

**Keywords:** autoimmune diseases, autoimmune polyglandular syndrome, autoimmune thyroiditis, celiac disease, micronutrient deficiencies

## Abstract

Celiac disease (CD) is an immune-mediated disorder frequently associated with autoimmune diseases (ADs) and nutritional deficiencies. This study aimed to determine the prevalence and spectrum of additional ADs and autoimmune polyglandular syndromes (APS), as well as micronutrient deficiencies, in adults with biopsy-confirmed CD. This prospective observational study included 102 adults with previously diagnosed, biopsy-confirmed CD who were under regular outpatient follow-up. Demographic and clinical characteristics, additional ADs, APS subtypes, laboratory parameters, micronutrient deficiencies, and adherence to a gluten-free diet assessed using the celiac dietary adherence test (CDAT) were evaluated. Additional ADs were identified in 26 of 102 patients (25.5%; 95% confidence interval [CI], 18.0–34.7), with autoimmune thyroiditis being the most frequent (12.7%; 95% CI, 7.6–20.6). APS was identified in 16 patients (15.7%; 95% CI, 9.9–24.0). APS type 3 was the most frequent subtype (11.8%; 95% CI, 6.9–19.4), followed by APS type 4 (2.0%; 95% CI, 0.5–6.9), whereas APS types 1 and 2 were each identified in 1.0% (95% CI, 0.2–5.3). At least 1 micronutrient deficiency was present in 93.1% (95% CI, 86.5–96.6), and 54.9% (95% CI, 45.2–64.2) had 2 or more deficiencies. Vitamin D deficiency was the most common (90.2%; 95% CI, 82.9–94.6), followed by folate deficiency (33.3%; 95% CI, 24.9–42.9), iron deficiency (29.4%; 95% CI, 21.4–38.9), vitamin B12 deficiency (17.6%; 95% CI, 11.5–26.2), and iron deficiency anemia (17.6%; 95% CI, 11.5–26.2). Patients with poor gluten-free diet adherence had significantly higher frequencies of 2 or more micronutrient deficiencies, folate deficiency, iron deficiency, vitamin B12 deficiency, and iron deficiency anemia than those with good adherence. Adults with established CD under follow-up had a substantial burden of additional ADs, defined APS phenotypes, and micronutrient deficiencies. Autoimmune thyroiditis and APS type 3 were the predominant autoimmune manifestations. Micronutrient deficiencies remained frequent even among patients with good self-reported gluten-free diet adherence, supporting continued nutritional monitoring during long-term follow-up.

## 1. Introduction

Celiac disease (CD) is a chronic immune-mediated systemic disorder triggered by dietary gluten in genetically susceptible individuals. Although CD primarily affects the small intestine, it is increasingly recognized as a multisystem disease with a broad spectrum of gastrointestinal and extraintestinal manifestations.^[1,2]^ Advances in serological testing and greater disease awareness have demonstrated that CD affects approximately 1% of the global population, making it a common immune-mediated disorder worldwide.^[3]^ Beyond intestinal injury and malabsorption, persistent immune dysregulation and shared genetic susceptibility predispose affected individuals to the development of additional autoimmune diseases (ADs).^[4,5]^

The coexistence of ADs in patients with CD has been well documented.^[5–7]^ Shared genetic factors, particularly HLA-DQ2 and HLA-DQ8 haplotypes, together with common immunological pathways, contribute to an increased risk of autoimmune thyroid disease, type 1 diabetes mellitus, autoimmune liver disease, Addison disease, and several other autoimmune conditions.^[4–7]^ Some of these disorders occur within the spectrum of autoimmune polyglandular syndrome (APS), a heterogeneous group of disorders characterized by the coexistence of multiple autoimmune endocrine and non-endocrine diseases.^[8,9]^ Because APS may remain clinically unrecognized until multiple autoimmune disorders become apparent, its early identification is important for timely surveillance, diagnosis, and long-term management of associated autoimmune conditions.^[8–10]^

Although previous studies have consistently demonstrated an increased prevalence of individual ADs among patients with CD, most investigations have focused on isolated autoimmune disorders, particularly autoimmune thyroid disease and type 1 diabetes mellitus, rather than evaluating APS or the broader AD spectrum.^[5,11,12]^ Consequently, the prevalence, subtype distribution, and clinical spectrum of APS in adults with biopsy-confirmed CD remain incompletely characterized.^[8,9]^ Furthermore, studies comprehensively evaluating APS together with the accompanying AD spectrum in biopsy-confirmed adult patients are limited.^[9,13]^ Better characterization of these autoimmune clusters may improve clinical recognition of patients at increased risk for multiple autoimmune disorders and facilitate more comprehensive long-term follow-up.

Therefore, the present prospective study aimed to determine the prevalence and clinical spectrum of APS in adults with biopsy-confirmed CD while comprehensively characterizing the spectrum of concomitant ADs. In addition, micronutrient deficiencies frequently encountered in CD were evaluated to provide a more comprehensive clinical characterization of this patient population.

## 2. Materials and methods

### 2.1. Study population

This prospective observational study included consecutive adult patients (≥18 years) with biopsy-confirmed CD who were recruited from the Internal Medicine Outpatient Clinic of Umraniye Training and Research Hospital, Istanbul, Türkiye, between December 1, 2021, and March 1, 2022. The diagnosis of CD was established by positive celiac-specific serology together with compatible duodenal histopathology in accordance with current international guidelines.^[14]^

Eligible participants had a previously established diagnosis of CD and were under regular outpatient follow-up at the time of enrollment. None of the participants were newly diagnosed during the study period. Patients with incomplete clinical or laboratory data and those with type 2 diabetes mellitus were excluded.

During the study period, 119 patients with CD were assessed for eligibility. Fourteen patients were excluded because of incomplete clinical or laboratory data, and 3 patients were excluded because of type 2 diabetes mellitus. Ultimately, 102 patients were included in the final analysis (Fig. 1).

**Figure 1.**
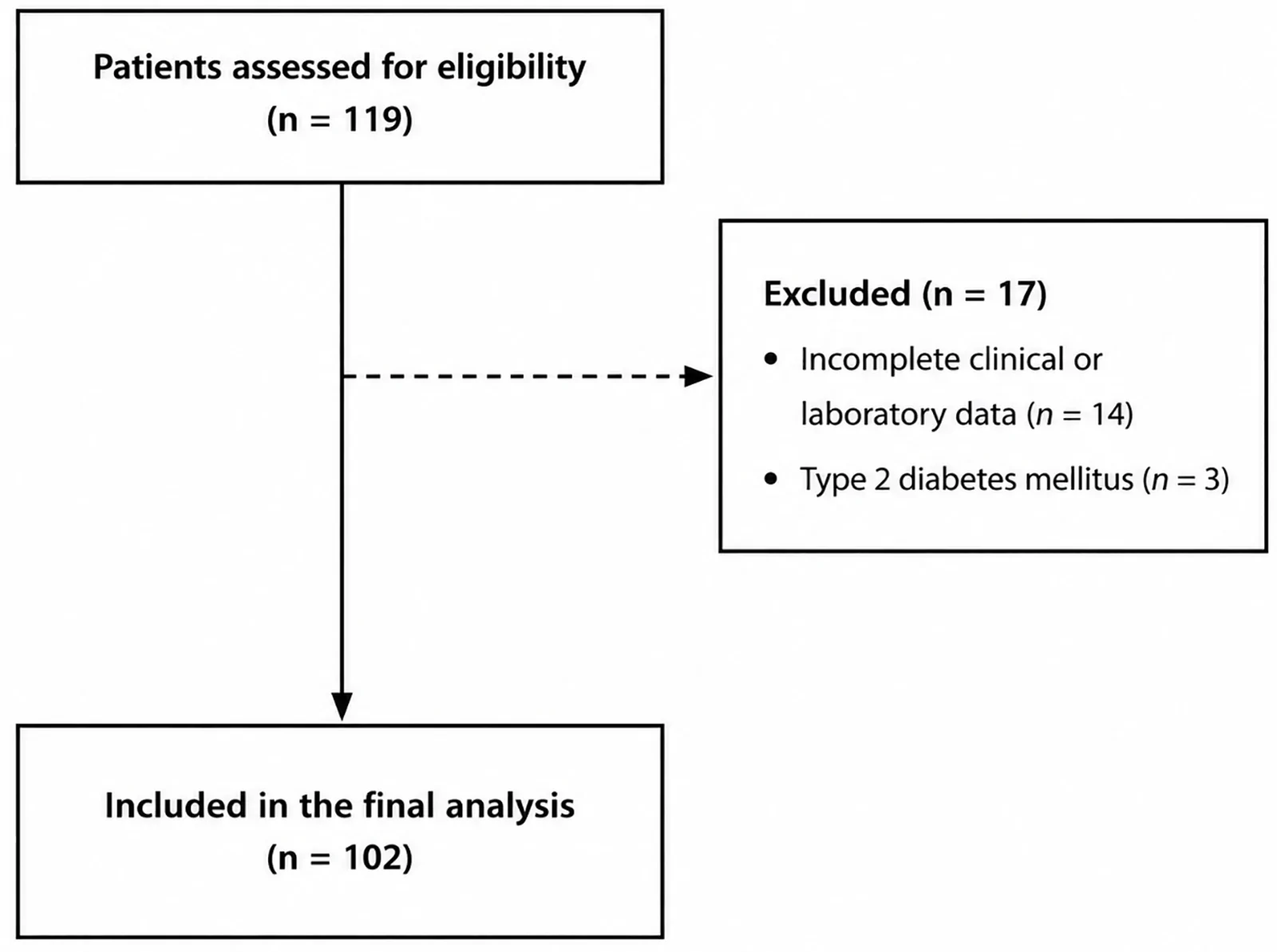
Flow diagram of patient selection. During the study period, 119 adults with biopsy-confirmed celiac disease were assessed for eligibility. Fourteen patients were excluded because of incomplete clinical or laboratory data, and 3 patients were excluded because of type 2 diabetes mellitus. Ultimately, 102 patients were included in the final analysis.

### 2.2. Clinical data collection

Demographic and clinical data were collected prospectively at the time of study enrollment through standardized patient interviews and review of electronic medical records. Demographic variables included age and sex. Clinical variables included the duration of symptoms before diagnosis, time since CD diagnosis, adherence to a gluten-free diet, and the presence of additional ADs.

Information on additional ADs was obtained from electronic medical records and verified through patient interviews. The diagnosis of each AD was based on previously established clinical, laboratory, histopathological, and imaging findings, as appropriate, according to accepted diagnostic criteria. To minimize potential sources of bias, consecutive eligible patients were prospectively enrolled, predefined inclusion and exclusion criteria were applied, and clinical and laboratory data were collected using standardized assessment procedures.

Adherence to a gluten-free diet was assessed using the validated celiac dietary adherence test (CDAT), a 7-item questionnaire specifically developed for patients with CD. Participants were classified as having good or poor adherence according to the validated CDAT cutoff, with scores <13 indicating good adherence and scores ≥13 indicating poor adherence.^[15]^ For analyses of nutritional status according to dietary adherence, participants were stratified into good-adherence (CDAT < 13) and poor-adherence (CDAT ≥ 13) groups.

### 2.3. Laboratory assessment

Venous blood samples were obtained from all participants during routine outpatient evaluation after an overnight fast. Laboratory analyses were performed in the central laboratory of Umraniye Training and Research Hospital using standard automated methods.

The laboratory evaluation included complete blood count, fasting serum glucose, serum albumin, iron, ferritin, transferrin saturation, vitamin B12, folate, 25-hydroxyvitamin D, calcium, phosphorus, parathyroid hormone, thyroid-stimulating hormone, free thyroxine, total immunoglobulin A (IgA), anti-tissue transglutaminase IgA, anti-endomysial antibody, anti-thyroid peroxidase antibody, and anti-thyroglobulin antibody. In patients with IgA deficiency, immunoglobulin G (IgG)-based celiac serology, including anti-tissue transglutaminase IgG and deamidated gliadin peptide IgG (DGP-IgG), was used for serological assessment.

Additional endocrine investigations, including morning serum cortisol measurements, were performed when clinically indicated.

### 2.4. Diagnostic definitions

APS was classified according to the established clinical classification.^[8,9]^ APS-1 was defined by the presence of at least 2 of the following major components: chronic mucocutaneous candidiasis, chronic hypoparathyroidism, and primary adrenal insufficiency. APS-2 was defined by the presence of primary adrenal insufficiency in combination with autoimmune thyroid disease and/or type 1 diabetes mellitus. APS-3 was defined by the coexistence of autoimmune thyroid disease with another AD, including CD, in the absence of primary adrenal insufficiency. APS-4 was defined by the coexistence of an autoimmune endocrine disease not fulfilling the criteria for APS-1, APS-2, or APS-3 with at least 1 additional AD.

The diagnosis of ADs was based on previously established clinical diagnoses documented in the medical records and supported by relevant clinical, laboratory, imaging, and histopathological findings according to accepted disease-specific diagnostic criteria. IgA deficiency was defined as a serum IgA concentration <7 mg/dL.

Micronutrient deficiencies were defined according to established international guideline recommendations.^[16–18]^ Vitamin D deficiency was defined as a serum 25-hydroxyvitamin D concentration <20 ng/mL; vitamin B12 deficiency as a serum vitamin B12 concentration <200 pg/mL; and folate deficiency as a serum folate concentration <4 ng/mL. Iron deficiency was defined as a serum ferritin concentration <15 ng/mL and/or transferrin saturation <20%. Anemia was defined according to World Health Organization criteria as a hemoglobin concentration <12 g/dL in women and <13 g/dL in men, and iron deficiency anemia was defined as anemia in the presence of iron deficiency. Hypocalcemia was defined as a serum total calcium concentration <8.5 mg/dL, and hypoalbuminemia as a serum albumin concentration <3.5 g/dL.

### 2.5. Outcome measures

The primary outcome was the prevalence and subtype distribution of APS in adults with biopsy-confirmed CD.

The secondary outcomes included the prevalence and spectrum of concomitant ADs and the frequency of micronutrient deficiencies, including iron deficiency anemia, vitamin B12 deficiency, folate deficiency, and vitamin D deficiency.

### 2.6. Statistical analysis

Statistical analyses were performed using IBM SPSS Statistics for Windows, version 25.0 (IBM Corp, Armonk). The distribution of continuous variables was assessed using the Kolmogorov–Smirnov and Shapiro–Wilk tests.

Continuous variables are presented as mean ± standard deviation, whereas categorical variables are presented as frequencies and percentages. Prevalence estimates are presented with 95% confidence intervals (CIs) calculated using the Wilson score method. Comparisons between 2 groups were performed using the independent-samples t-test or the Mann–Whitney *U* test, depending on data distribution. Comparisons among multiple groups were performed using 1-way analysis of variance or the Kruskal–Wallis test, as appropriate. The prevalence of micronutrient deficiencies was additionally compared between patients with good (CDAT < 13) and poor (CDAT ≥ 13) adherence to a gluten-free diet. Categorical variables were compared using the chi-square test or Fisher’s exact test, as appropriate.

All statistical tests were two-sided, and a *P* value < .05 was considered statistically significant.

### 2.7. Ethics

The study protocol was approved by the Clinical Research Ethics Committee of Umraniye Training and Research Hospital (approval date: November 18, 2021; approval number: 332). Written informed consent was obtained from all participants before enrollment. The study was conducted in accordance with the ethical principles of the Declaration of Helsinki.

## 3. Results

### 3.1. Baseline characteristics

A total of 102 adults with biopsy-confirmed CD were included in the final analysis. The baseline demographic, clinical, and laboratory characteristics of the study population are presented in Table 1. The mean age of the participants was 36.3 ± 11.6 years, and the majority were women (80.4%). The mean disease duration was 6.3 ± 5.8 years. Overall, the study population demonstrated preserved nutritional status, with a mean serum albumin concentration of 4.3 ± 0.4 g/dL and a mean serum IgA concentration of 236.8 ± 92.4 mg/dL. IgA deficiency was identified in 1 of 102 patients (1.0%). Mean serum concentrations of folate, vitamin B12, and 25-hydroxyvitamin D were 7.3 ± 4.5 ng/mL, 374.2 ± 149.6 pg/mL, and 18.3 ± 9.2 ng/mL, respectively.

**Table 1:** Baseline demographic, clinical, and laboratory characteristics of the study population.

Characteristic	Overall (n = 102)
Demographic characteristics	
Age, yr	36.3 ± 11.6
Female sex, n (%)	82 (80.4)
Body mass index, kg/m^2^	23.8 ± 3.9
Clinical characteristics	
Disease duration, yr	6.3 ± 5.8
Duration of gluten-free diet, yr	5.2 ± 4.8
Good adherence to gluten-free diet, n (%)	67 (65.7)
Selected laboratory characteristics	
Hemoglobin, g/dL	13.4 ± 1.6
Ferritin, ng/mL	41.8 ± 36.5
Albumin, g/dL	4.3 ± 0.4
Immunoglobulin A, mg/dL	236.8 ± 92.4
Thyroid-stimulating hormone, mIU/L	2.48 ± 1.74
Free thyroxine, ng/dL	1.18 ± 0.19
Folate, ng/mL	7.3 ± 4.5
Vitamin B12, pg/mL	374.2 ± 149.6
25-hydroxyvitamin D, ng/mL	18.3 ± 9.2

Data are presented as mean ± standard deviation (SD) or n (%), as appropriate.

### 3.2. Autoimmune diseases and autoimmune polyglandular syndrome

Additional ADs were identified in 26 of 102 patients (25.5%), including 3 patients (2.9%) with 2 or more additional ADs. Autoimmune thyroiditis was the most frequently observed additional AD, affecting 13 patients (12.7%). Other additional ADs included Addison disease, chronic hypoparathyroidism, and psoriasis (3 patients each, 2.9%), type 1 diabetes mellitus and vitiligo (2 patients each, 2.0%), and ankylosing spondylitis, Behçet disease, and Sjögren syndrome (1 patient each, 1.0%) (Table 2).

**Table 2:** Additional ADs and APS in the study population.

Characteristic	n (%)	95% CI
Additional autoimmune disease burden		
Patients with ≥1 additional AD	26 (25.5)	18.0–34.7
Patients with ≥2 additional ADs	3 (2.9)	1.0–8.3
Individual additional autoimmune diseases		
Autoimmune thyroiditis	13 (12.7)	7.6–20.6
Addison disease	3 (2.9)	1.0–8.3
Chronic hypoparathyroidism	3 (2.9)	1.0–8.3
Psoriasis	3 (2.9)	1.0–8.3
Type 1 diabetes mellitus	2 (2.0)	0.5–6.9
Vitiligo	2 (2.0)	0.5–6.9
Ankylosing spondylitis	1 (1.0)	0.2–5.3
Behçet disease	1 (1.0)	0.2–5.3
Sjögren syndrome	1 (1.0)	0.2–5.3
Autoimmune polyglandular syndrome		
Any APS	16 (15.7)	9.9–24.0
APS type 1	1 (1.0)	0.2–5.3
APS type 2	1 (1.0)	0.2–5.3
APS type 3	12 (11.8)	6.9–19.4
APS type 4	2 (2.0)	0.5–6.9

Data are presented as n (%) with 95% confidence intervals (CIs). Percentages and 95% CIs were calculated using the total study population (N = 102) as the denominator. Individual autoimmune diseases were not mutually exclusive; therefore, their frequencies do not sum to the number of patients with ≥1 additional autoimmune disease. APS was classified according to the predefined diagnostic criteria. The 95% CIs for proportions were calculated using the Wilson score method.

APS = Autoimmune polyglandular syndrome.

APS was diagnosed in 16 patients (15.7%). APS type 3 was the most frequent subtype, accounting for 12 patients (11.8%), followed by APS type 4 in 2 patients (2.0%). APS type 1 and APS type 2 were each identified in 1 patient (1.0%). The distribution of AD combinations according to APS subtype is presented in Table 3.

**Table 3:** Autoimmune disease combinations according to APS subtype.

APS subtype	Autoimmune disease combination	n (%)
APS type 1	Celiac disease + Addison disease + chronic hypoparathyroidism + chronic mucocutaneous candidiasis	1 (1.0)
APS type 2	Celiac disease + autoimmune thyroiditis + Addison disease	1 (1.0)
APS type 3	Celiac disease + autoimmune thyroiditis	12 (11.8)
APS type 4	Celiac disease + type 1 diabetes mellitus	2 (2.0)

Data are presented as n (%), calculated using the total study population (N = 102) as the denominator.

APS = Autoimmune polyglandular syndrome, CD = celiac disease.

### 3.3. Nutritional deficiencies

Overall, 95 patients (93.1%) had at least 1 micronutrient deficiency, whereas 56 (54.9%) and 23 (22.5%) had 2 or more and 3 or more concomitant micronutrient deficiencies, respectively. Vitamin D deficiency was the most prevalent nutritional abnormality, affecting 92 patients (90.2%), followed by folate deficiency (33.3%), iron deficiency (29.4%), vitamin B12 deficiency (17.6%), and iron deficiency anemia (17.6%). Hypocalcemia and hypoalbuminemia were observed in 11 (10.8%) and 7 (6.9%) patients, respectively. The most frequent concomitant deficiencies were vitamin D deficiency combined with folate deficiency (30.4%) and iron deficiency (26.5%) (Table 4).

**Table 4:** Nutritional status and micronutrient deficiencies in the study population.

Nutritional parameter	n (%)	95% CI
Overall nutritional burden		
Patients with ≥1 micronutrient deficiency	95 (93.1)	86.5–96.6
Patients with ≥2 micronutrient deficiencies	56 (54.9)	45.2–64.2
Patients with ≥3 micronutrient deficiencies	23 (22.5)	15.5–31.6
Individual micronutrient deficiencies		
Vitamin D deficiency	92 (90.2)	82.9–94.6
Folate deficiency	34 (33.3)	24.9–42.9
Iron deficiency	30 (29.4)	21.4–38.9
Vitamin B12 deficiency	18 (17.6)	11.5–26.2
Iron deficiency anemia	18 (17.6)	11.5–26.2
Hypocalcemia	11 (10.8)	6.1–18.3
Hypoalbuminemia	7 (6.9)	3.4–13.5
Most frequent concomitant deficiencies		
Vitamin D deficiency + folate deficiency	31 (30.4)	22.3–39.9
Vitamin D deficiency + iron deficiency	27 (26.5)	18.9–35.8
Vitamin D deficiency + vitamin B12 deficiency	17 (16.7)	10.7–25.1
Iron deficiency + vitamin B12 deficiency	9 (8.8)	4.7–15.9

Data are presented as n (%) with 95% confidence intervals (CIs). Percentages and 95% CIs were calculated using the total study population (N = 102) as the denominator. Micronutrient deficiencies were defined according to the predefined diagnostic criteria described in the Methods section. Individual deficiencies were not mutually exclusive; therefore, patients may have had more than 1 micronutrient deficiency. The 95% CIs for proportions were calculated using the Wilson score method.

According to the CDAT, 67 patients (65.7%) had good adherence to a gluten-free diet, and 35 (34.3%) had poor adherence. Patients with poor adherence had a higher prevalence of 2 or more micronutrient deficiencies than those with good adherence (71.4% vs 46.3%, *P* = .015), as well as 3 or more deficiencies (37.1% vs 14.9%, *P* = .011). Folate deficiency (48.6% vs 25.4%, *P* = .018), iron deficiency (42.9% vs 22.4%, *P* = .031), vitamin B12 deficiency (28.6% vs 11.9%, *P* = .036), and iron deficiency anemia (28.6% vs 11.9%, *P* = .036) were also more frequent among patients with poor adherence. In contrast, the prevalence of at least 1 micronutrient deficiency, vitamin D deficiency, hypocalcemia, and hypoalbuminemia did not differ significantly between the adherence groups (Table 5).

**Table 5:** Micronutrient deficiencies according to gluten-free diet adherence assessed by the celiac dietary adherence test.

Nutritional parameter	Good adherence (CDAT < 13), n = 67 n (%)	Poor adherence (CDAT ≥ 13), n = 35 n (%)	*P* value
Overall nutritional burden			
≥1 micronutrient deficiency	60 (89.6)	35 (100.0)	.092
≥2 micronutrient deficiencies	31 (46.3)	25 (71.4)	.015
≥3 micronutrient deficiencies	10 (14.9)	13 (37.1)	.011
Individual micronutrient deficiencies			
Vitamin D deficiency	58 (86.6)	34 (97.1)	.158
Folate deficiency	17 (25.4)	17 (48.6)	.018
Iron deficiency	15 (22.4)	15 (42.9)	.031
Vitamin B12 deficiency	8 (11.9)	10 (28.6)	.036
Iron deficiency anemia	8 (11.9)	10 (28.6)	.036
Hypocalcemia	6 (9.0)	5 (14.3)	.505
Hypoalbuminemia	4 (6.0)	3 (8.6)	.689

Data are presented as n (%) within each gluten-free diet adherence group. Good adherence was defined as a CDAT score <13 and poor adherence as a CDAT score ≥13. P values were calculated using the chi-square test or Fisher’s exact test, as appropriate.

CDAT = celiac dietary adherence test.

## 4. Discussion

In this prospective study of adults with biopsy-confirmed CD, additional ADs were common, with autoimmune thyroiditis representing the predominant associated condition. APS was identified in 15.7% of the study population, with type 3 representing the predominant subtype. In addition, nutritional deficiencies were highly prevalent, particularly vitamin D deficiency, and multiple concomitant micronutrient deficiencies were frequently observed. Collectively, these findings demonstrate that the clinical burden of CD extends beyond intestinal involvement and includes a substantial burden of additional ADs and nutritional deficiencies, highlighting the importance of comprehensive evaluation and long-term multidisciplinary follow-up.

Additional ADs were identified in 25.5% of patients. This prevalence is consistent with previous studies demonstrating that autoimmune comorbidities are common in adults with CD, although substantial variability has been reported according to study design, patient characteristics, duration of gluten exposure, and the diagnostic criteria used for ADs.^[6,19]^ Autoimmune thyroiditis was the most frequent additional AD, affecting 12.7% of patients, consistent with the well-established association between CD and autoimmune thyroid disease.^[11,19]^ This association is thought to reflect shared genetic susceptibility, particularly HLA-DQ2/DQ8 haplotypes, together with common immunological mechanisms involving loss of immune tolerance and persistent immune activation.^[5,20]^

Other autoimmune conditions were less frequent in the present cohort. Type 1 diabetes mellitus and vitiligo were each identified in 2.0% of patients, whereas ankylosing spondylitis was identified in 1.0%. The coexistence of CD with type 1 diabetes mellitus is well recognized and is supported by shared genetic and immune-mediated susceptibility.^[21]^ An association between CD and vitiligo has also been described, although findings across studies have not been entirely consistent.^[22]^ Emerging evidence additionally suggests shared genetic and immune-related mechanisms between CD and ankylosing spondylitis.^[23]^ However, given the very small numbers of patients with these individual conditions in the present cohort, these findings should be interpreted descriptively and do not permit disease-specific conclusions. Psoriasis, Addison disease, chronic hypoparathyroidism, Sjögren syndrome, and Behçet disease were also observed at low frequencies, further illustrating the heterogeneous spectrum of autoimmune comorbidity in CD.

APS was identified in 15.7% of patients, with type 3 representing the most frequent subtype. This finding is consistent with previous studies demonstrating the importance of autoimmune thyroid disease within the autoimmune spectrum of CD and APS. However, most previous investigations have primarily focused on overall APS prevalence or individual autoimmune disorders rather than the distribution of APS subtypes and accompanying AD combinations.^[9,13,24]^ The systematic classification of APS according to established diagnostic criteria, together with comprehensive characterization of the associated autoimmune phenotypes, provides additional insight into the clinical spectrum of APS in adults with CD.^[8,25]^ The predominance of APS type 3 reflects the contribution of autoimmune thyroiditis to the autoimmune spectrum of CD, whereas the lower frequency of APS type 4 illustrates that only a subset of patients with additional ADs fulfilled the criteria for this APS subtype.^[9,26]^ Furthermore, detailed characterization of AD combinations may facilitate recognition of APS and support individualized surveillance strategies in patients with CD.^[13,25,26]^ Collectively, these findings suggest that the evaluation of APS in CD should extend beyond prevalence estimates to include detailed phenotypic characterization, thereby supporting a more comprehensive approach to clinical assessment and long-term patient management.^[8,25]^ Because the study population consisted of patients with previously diagnosed CD who were already under outpatient follow-up, the observed frequencies of ADs and APS should be interpreted as prevalence estimates within an established CD cohort rather than as estimates at the time of initial CD diagnosis. Patients with longer disease duration and follow-up may also have had greater opportunity for associated autoimmune conditions to be recognized.

IgA deficiency was identified in 1 patient (1.0%) in the present cohort. The recognized association between IgA deficiency and CD, potentially related in part to shared human leukocyte antigen susceptibility, represents an important consideration in the serological evaluation of patients with CD. Because IgA deficiency may reduce the diagnostic reliability of IgA-based serological tests, assessment of total serum IgA is important when interpreting celiac-specific serology. In patients with IgA deficiency, the use of IgG-based serological markers provides an alternative approach for serological assessment. Accordingly, evaluation of IgA status together with appropriate selection of serological tests may help reduce the risk of false-negative serological findings in this subgroup.^[27,28]^

Nutritional deficiencies were highly prevalent, with at least 1 micronutrient deficiency identified in 93.1% of patients and vitamin D deficiency representing the most common abnormality (90.2%), followed by folate deficiency, iron deficiency, and vitamin B12 deficiency. These findings are consistent with previous studies demonstrating that micronutrient deficiencies remain common in adults with CD despite increasing awareness and earlier diagnosis.^[29,30]^ Vitamin D deficiency was particularly frequent in the present cohort (90.2%). Previous evidence indicates that vitamin D deficiency is common in CD and may reflect several factors, including impaired intestinal absorption and nutritional characteristics of a gluten-free diet.^[31,32]^ Notably, a large global analysis of population-based studies reported that 47.9% of the general population had serum 25-hydroxyvitamin D concentrations below 50 nmol/L (20 ng/mL), which corresponds to the threshold used to define vitamin D deficiency in the present study.^[33]^ Importantly, participants in the present study were enrolled between December and March, predominantly during the winter season, which may have contributed to the particularly high prevalence observed. Therefore, the high frequency of vitamin D deficiency in this cohort should not be attributed solely to CD-related malabsorption. Similarly, deficiencies of iron, folate, and vitamin B12 likely reflect the combined effects of disease-related malabsorption, delayed diagnosis, and variable adherence to a gluten-free diet.^[29,34,35]^ More than half of the patients had multiple concomitant micronutrient deficiencies, emphasizing that nutritional abnormalities in CD frequently coexist rather than occur in isolation.^[29,30]^ The stratified analysis according to gluten-free diet adherence provides further insight into these findings. Patients with poor adherence had significantly higher frequencies of multiple micronutrient deficiencies, with both ≥2 and ≥3 concomitant deficiencies being more common than among patients with good adherence. Folate deficiency, iron deficiency, vitamin B12 deficiency, and iron deficiency anemia were also significantly more frequent in the poor-adherence group. These findings support an association between poorer adherence to a gluten-free diet and a greater burden of selected micronutrient deficiencies. Nevertheless, micronutrient abnormalities remained frequent even among patients with good adherence; notably, 89.6% had at least 1 deficiency and 86.6% had vitamin D deficiency. This observation suggests that dietary adherence alone may not fully account for the nutritional burden in treated CD. The CDAT primarily assesses adherence to a gluten-free diet and does not directly evaluate overall dietary quality or micronutrient adequacy; therefore, nutritional deficiencies may persist despite apparently good adherence. These findings underscore the importance of comprehensive nutritional assessment at diagnosis and regular monitoring during long-term follow-up to facilitate timely identification and correction of micronutrient deficiencies.^[14,36]^

The present study has several important strengths. To our knowledge, this is among the few prospective studies to comprehensively evaluate additional ADs, APS subtypes, and nutritional deficiencies simultaneously in adults with biopsy-confirmed CD. The systematic classification of APS according to established diagnostic criteria, together with the detailed characterization of accompanying AD combinations, provides further insight into the autoimmune spectrum associated with CD. Furthermore, the prospective study design, standardized clinical and laboratory assessments, and the use of a validated instrument (CDAT) to assess adherence to a gluten-free diet enhance the methodological rigor of the study.

Nevertheless, several limitations should be acknowledged. First, this was a single-center study with a moderate sample size, which may limit the generalizability of these findings. Second, although data were collected prospectively, the observational design precludes conclusions regarding causal relationships between CD and associated autoimmune conditions. In addition, the study included a prevalent cohort of previously diagnosed CD patients under regular follow-up, which may have introduced selection and prevalence-related biases and may limit the generalizability of the findings to newly diagnosed or unselected CD populations. Patients with longer follow-up may have had a greater opportunity for associated ADs to be detected. Moreover, the timing of AD and APS diagnoses relative to the diagnosis of CD was not systematically recorded; therefore, the temporal sequence between CD and associated autoimmune conditions could not be evaluated. Third, Marsh classification data were unavailable, precluding evaluation of potential associations between histological severity and autoimmune or nutritional manifestations. In addition, although adherence to a gluten-free diet was assessed using the validated CDAT, this instrument primarily evaluates gluten-free dietary adherence rather than overall diet quality or micronutrient adequacy. Therefore, good adherence as defined by the CDAT does not necessarily indicate a nutritionally adequate diet. Furthermore, because participant enrollment occurred predominantly during the winter months, seasonal variation may have contributed to the high prevalence of vitamin D deficiency. Information regarding vitamin D supplementation was not systematically collected; therefore, supplementation status could not be incorporated into the analysis.

## 5. Conclusion

In this prospective study of adults with biopsy-confirmed CD, autoimmune comorbidity and nutritional deficiencies represented 2 prominent and clinically relevant components of the disease spectrum. Autoimmune thyroiditis was the most common associated AD, while APS type 3 was the predominant APS subtype. Importantly, additional AD did not necessarily indicate the presence of APS, underscoring the importance of distinguishing individual autoimmune comorbidities from defined APS phenotypes. Nutritional deficiencies were also remarkably common, particularly vitamin D deficiency, and a greater micronutrient deficiency burden was observed among patients with poorer gluten-free diet adherence. Taken together, these findings emphasize that the clinical assessment of CD should extend beyond gastrointestinal manifestations to encompass both its autoimmune phenotype and nutritional burden during long-term follow-up.

## Author contributions

**Conceptualization:** Senem Kuşaksızoğlu Kara, Sema Basat.

**Data curation:** Senem Kuşaksızoğlu Kara.

**Formal analysis:** Senem Kuşaksızoğlu Kara, Çağdaş Kara.

**Investigation:** Senem Kuşaksızoğlu Kara.

**Methodology:** Senem Kuşaksızoğlu Kara, Sema Basat, Çağdaş Kara.

**Supervision:** Sema Basat.

**Validation:** Çağdaş Kara.

**Writing – original draft:** Senem Kuşaksızoğlu Kara.

**Writing – review & editing:** Senem Kuşaksızoğlu Kara, Sema Basat, Çağdaş Kara.
